# Young-onset gastric cancer and Epstein–Barr Virus (EBV) – a major player in the pathogenesis?

**DOI:** 10.1186/s12885-020-6517-0

**Published:** 2020-01-14

**Authors:** Assaf Moore, Elad Hikri, Tal Goshen-Lago, Tamar Barkan, Sara Morgenstern, Elena Brook, Annett Maderer, Wilfried Roth, Noa Gordon, Hanoch Kashtan, Baruch Brenner, Markus Moehler, Irit Ben Aharon

**Affiliations:** 1 Institute of Oncology, Davidoff Cancer Center, Rabin Medical Center, Ze’ev Jabotinsky Rd 39, 4941492 Petah Tikva, Israel; 2 Sackler Faculty of Medicine, Tel Aviv University, Ramat Aviv, 6997801 Tel Aviv, Israel; 3 Department of Pathology, Rabin Medical Center, Ze’ev Jabotinsky Rd 39, 4941492 Petah Tikva, Israel; 4 Department of Internal Medicine I, University Medical Center of the Johannes Gutenberg University Mainz, Langenbeckstraße 1, 55131 Mainz, Germany; 5 Tissue Bank and Institute of Pathology, University Medical Center of the Johannes Gutenberg University Mainz, Langenbeckstraße 1, 55131 Mainz, Germany; 6 Department of surgery B, Beilinson campus, Rabin Medical Center, Ze’ev Jabotinsky Rd 39, 4941492 Petah Tikva, Israel

**Keywords:** Gastric cancer, Young patients, Epstein-Barr virus (EBV), MSI, PD-L1

## Abstract

**Objective:**

Gastric cancer (GC) is a leading cause of cancer death, occurs predominantly in older age, with increasing incidence in young patients. The Cancer Genome Atlas indicates four subtypes for GC among which Epstein-Barr virus (EBV) subtype is estimated at 8.7%. We aim to determine the prevalence of EBV subtype in young GC patients (≤45 years) compared with an average-onset cohort (≥55 years) and characterize the clinicopathologic pattern of young-onset GC.

**Methods:**

Gastric cancer samples of patients of both cohorts were screened for EBV by qPCR. Additional staining was done for Human epidermal growth factor receptor 2 (HER2), microsatellite instability (MSI) status and Programmed death-ligand 1 (PD-L1). Demographics and clinical data were retrieved from the medical records.

**Results:**

Thirty-nine young-onset and 35 average-onset GC patients were reviewed. There was no apparent difference in tumor location, family history, histology and HER2 status between the cohorts. More young-onset patients were diagnosed with metastatic disease (27% vs 9%, *p* = 0.0498). EBV was significantly more prevalent in the young-onset cohort (33% vs 11%, *p* = 0.025). 15/17 EBV positive patients were under the median age of diagnosis for GC in the US (68 years). MSI-H was found only in the average-onset cohort [0% vs 27%, *p* = 0.001). PD-L1 positivity was higher in the young-onset cohort (31% vs 3%, *p* = 0.002).

**Conclusion:**

Our study indicates that EBV subtype is more prevalent in young-onset GC and may play a key role in the pathogenesis. Higher rate of PD-L1 positivity in young-onset GC could change treatment strategies. We are currently evaluating these findings in a prospective trial.

## Background

Gastric cancer (GC) is the third and fifth most common cause of cancer related death in the world in males and females, respectively [1, 2]. Former data have shown that GC rates are twice as high in men, GC occurs predominantly in older age groups [2]. The median age at diagnosis in the United States is 68 years [3]. Several studies have indicated a trend toward steadily increasing incidence of GC in young patients over the past few decades [4]. Patients aged 40 years or younger comprised 4.6 to 6.2% of GC incidence [4, 5]. Former studies described the clinicopathological features of GC in young patients and implied that they were different from those of older patients [4, 5]. Gastric carcinogenesis is thought to be associated with multiple environmental and genetic factors. Although the etiology of GC not fully understood, infectious agents have been recognized to participate in neoplastic transformation [1, 2]. Helicobacter pylori (*H. pylori*) is the major causative agent of GC [1, 2].

The Epstein-Barr virus (EBV) is a double-stranded DNA γ-herpes virus, and has been associated with both lymphoid and epithelial malignancies since first discovered in tumor cells of Burkitt’s lymphoma in 1964 [6]. Approximately 90% of adults will have antibodies for EBV. During the latent phase of infection, EBV nuclear antigens (EBNAs) and latent membrane proteins (LMPs) are expressed in infected cells. EBNAs and LMPs play a role in the process of cell immortalization [6]. A Meta-analysis has found that the overall prevalence of EBV positivity in GC is estimated at 8.7% [7]. There is 2-fold difference by sex: 11.1% of cases in males, vs 5.2% in females. EBV positivity is found in about 13% of tumors arising in the gastric cardia or corpus and in 5.2% of antral tumors (*p* < 0.01) [7]. There was no difference between intestinal and diffuse histologies [7]. In an analysis of 8336 patients with GC, the presence of EBV had a favorable impact on GC patient’s survival [8] whereas the pooled HR for OS in GC patients was 0.67 (95% CI: 0.55–0.79; *p* < 0.001) [8]. One suggested explanation for this finding is that EBV infection recruits lymphocytes which induce an immune cell infiltration into tumor areas [6, 8]. The Cancer Genome Atlas (TCGA) project proposed a molecular classification of gastric cancer into four subtypes: tumors positive for EBV which display recurrent Phosphatidylinositol-4,5-Bisphosphate 3-Kinase Catalytic Subunit Alpha (PIK3CA) mutations, extreme DNA hypermethylation, and amplification of Janus kinase 2 (JAK2), cluster of differentiation 274 (CD274) (also known as Programmed death-ligand 1, PD-L1) and Programmed cell death 1 ligand 2 (PDCD1LG2) (also known as Programmed cell death ligand 2, PD-L2); microsatellite unstable tumors; genomically stable tumors; and tumors with chromosomal instability [9]. The prevalence of the EBV subtype in this reported population was 9%.

The differential prevalence of EBV positive GC subtype among young-onset GC is unknown. In this study, we hypothesized that the EBV subtype may be more prevalent in the young-onset GC cohort and therefore aimed to determine the prevalence of EBV positivity in young GC patients compared with an older cohort, as well as to characterize the clinical-pathological characteristics in these two age-distinct cohorts.

## Materials and methods

### Patients

The study has been approved by the local institutional review board committees of both medical centers.

All consecutive young-onset (age < 45 years) GC patients allocated from the Davidoff Center database, and a control cohort of consecutive average-age onset controls (age > 55 years) diagnosed and treated in Davidoff Center, between 1997 and 2016, for whom a pathological sample could be obtained were included in the study. Additional cohort of consisted of consecutive young-onset GC patients treated at the University Medical Center of the Johannes Gutenberg University Mainz, Germany, between 2006 and 2013 for whom a pathological sample could be obtained.

### Tissue analysis

All cases were reviewed by pathologist who identified the tumor in the block and guided the preparation.

### Detection of EBV by PCR

Genomic DNA was extracted from formalin-fixed, paraffin-embedded (FFPE) tissues using ReliaPrep™ FFPE gDNA Miniprep System (promega; # A2351, Madison, WI, USA) according to manufacturer protocol.

EBNA3A primers that were used for the detection of positive-EBV samples by PCR were: 5′-GAAACCAAGACCAGAGGTCC-3′ and 5′-TCCCAGGGCCGGACAATAGG-3′ [10]. Control GAPDH primers were 5’CAAGGTCATCCATGACAACTTTG-3′5’- GGGCCATCCACAGTCTTCTG − 3′.

### Molecular characterization

Human epidermal growth factor receptor 2 (HER2) status was detected using immunohistochemistry (IHC) staining with Her2 antibody (cat. # PATHWAY anti-HER-2/neu (4B5), Ventana, Tucson, AZ, USA). Standard American Society of Clinical Oncology / College of American Pathologists ASCO/CAP IHC classification for HER2 positivity (0, 1+, 2+ and 3+) was used. In cases were IHC results were considered equivocal (HER2 overexpression 2+), fluorescent in situ hybridization (FISH) analysis was performed. Evaluation of mismatch repair (MMR) protein expression was performed using antibodies to MutL homolog 1 (MLH1) (cat. no.# 790–4535, Ventana, PMS1 homolog 2, mismatch repair system component (PMS2) (cat. no. #760–4531, Ventana, Tucson, AZ, USA), mutS homolog 6 (MSH6) (cat. no. #287R-26, Cell Marque, Rocklin, CA, USA) and MutS protein homolog 2 (MSH2) (cat. no. # G219–1129, Cell Marque, Rocklin, CA, USA). Loss of MMR protein expression defined the patient as microsatellite instability high (MSI-H). PD-L1 expression was determined using IHC staining procedure with Dako Autostainer Link 48 platform (Agilent, Santa Clara, CA, USA) and an automated staining protocol validated for the PD-L1 IHC 22C3 pharmDx assay (Agilent). PD-L1 scoring was done according to FDA approved scoring guideline for PD-L1 in GC.

### Clinical data

Patient demographics including age, sex, smoking and family history, as well as clinico-pathological features such as tumor location, histology and presence of *H. pylori* were extracted from medical records. The date of diagnosis was known for all patients, and survival was calculated by time of death or date of last follow-up when appropriate.

### Statistics

Data was analyzed using the Statistical Package for the Social Sciences 22.0 (SPSS) at a significance level of 0.05. χ2 test was used for categorical data, and the Mann–Whitney-U test or Student’s T test were used for continuous data. Survival have been modeled using the Kaplan-Meier method.

## Results

### Patients and clinical characteristics

Thirty-nine young-onset GC patients (27 from the Davidoff Cancer Center, 12 from the University Medical Center of the Johannes Gutenberg University Mainz) and 35 average-onset GC patients (all from the Davidoff Cancer Center) were included in the study. The clinical characteristics are summarized in Table 1. The median age for the young-onset was 40 years (range 21–45) and for the average-onset 69 years (range 50–90). There appeared to be no differences between the age groups and between the German and Israeli cohorts in terms of sex, tumor location, smoking history, *H. pylori* positivity, diffuse subtype and signet ring cell variant. Family history of malignancy was known for the Israeli patients only, and there was no difference in its prevalence between the age groups (*p* = 0.38). While there was no variation in the overall stage distribution, the proportion of patients with metastatic disease (stage IV) was 9 and 27% in the average and young-onset cohort, respectively (*p* < 0.05). There was no difference in the proportion of patients undergoing curative resection (85% vs 80%, *p* = 0.15) or in disease free survival (DFS) (161.3 vs 145.2 months, *p* = 0.75) between the the average and young-onset cohorts. There was no difference in the median number of systemic treatment lines for metastatic disease between the average and young-onset cohorts (1 vs 1.5, respectively, *p* = 0.15).

**Table 1:** Patient Characteristics

Factor	Average Onset *n* = 35	Young Onset *n* = 39	*p*-value
Male sex, n(%)	20 (57%)	17 (43%)	NS
Family history of malignancy, n(%)	13 (48%)	13 (37%)	NS
Smoking history, n(%)	18 (49%)	18 (49%)	NS
HPpositive, n(%)	7 (47%)	8 (36%)	NS
Proximal tumor location, n(%)	11 (32%)	7 (22%)	NS
Signet ring cell, n(%)	13 (37%)	18 (55%)	NS
Stage IV at diagnosis, n(%)	3 (9%)	9 (27%)	*P* < 0.05
HER2 positive, n(%)	3 (9%)	2 (5%)	NS
EBV positive, n(%)	13 (33%)	4 (11%)	*P* < 0.05
Median OS, months	47.8 months	69.7 months	NS

*Abbreviations*: *HP* Helicobacter Pylori, *HER2* Human epidermal growth factor receptor 2, *EBV* Epstein–Barr Virus, *OS* overall survival

Data was missing on smoking history (5 patients), *H. pylori* (37), tumor location (13), HER2 (8), stage at diagnosis (5)

### Molecular characteristics

#### EBV

EBV status was known for all patients, and 17 patients (23%) were found to positive. The EBV subtype was significantly more prevalent in the young-onset cohort 13 (33%) compared with 4 (11%) patients in the average-onset (*p* < 0.05). There was no difference between the Israeli and the German young-onset cohort in the rate of EBV positivity.

#### MSI

**Fig. 1:**
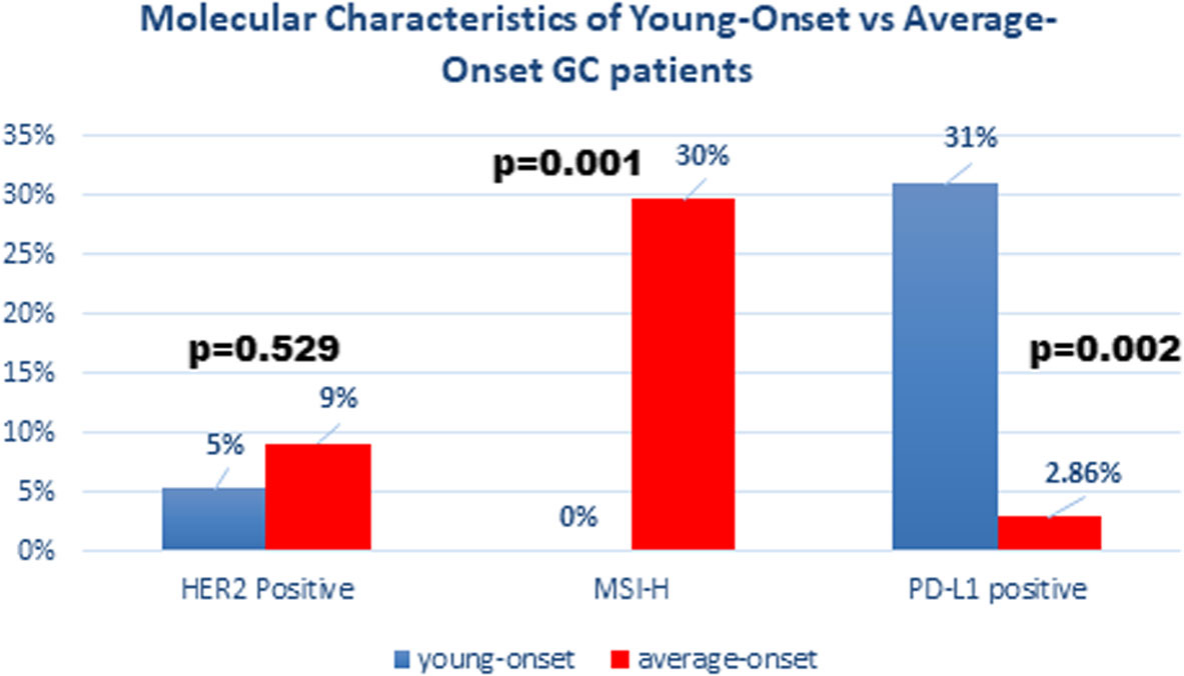
Molecular Characteristics of Young-Onset vs Average-Onset GC Patients

MSI status was available for 62 patients. MSI-H was only found in the average-onset cohort – 8/27, 30% vs 0% in the young-onset (*p* < 0.05) (Fig. 1). All MSI-H tumors were EBV and PD-L1 negative. Six patients had loss of MLH1 & PMS2, one patient loss of MSH6 & MSH2, and one patient had loss of MLH1, MSH6, MSH2 &PMS2.

#### HER2

HER2 status was available in 71 patients. HER2 was positive in 9% of average-onset patients compared with 5% in the young-onset (*p* = 0.53) (Fig. 1). All EBV positive cases were HER2 negative (0/17), compared with an overall prevalence of 9% (5/56) of HER2 positivity in EBV negative cases (*p* = 0.23). One HER2 positive patient was PD-L1 positive.

#### PD-L1

PDL-1 status was available for all patients. Twelve of 39 (31%) young-onset patients were PD-L1 positive, compared with 1/35 (3%) of average-onset patients (*p* < 0.05) (Fig. 1).

### Survival

**Fig. 2:**
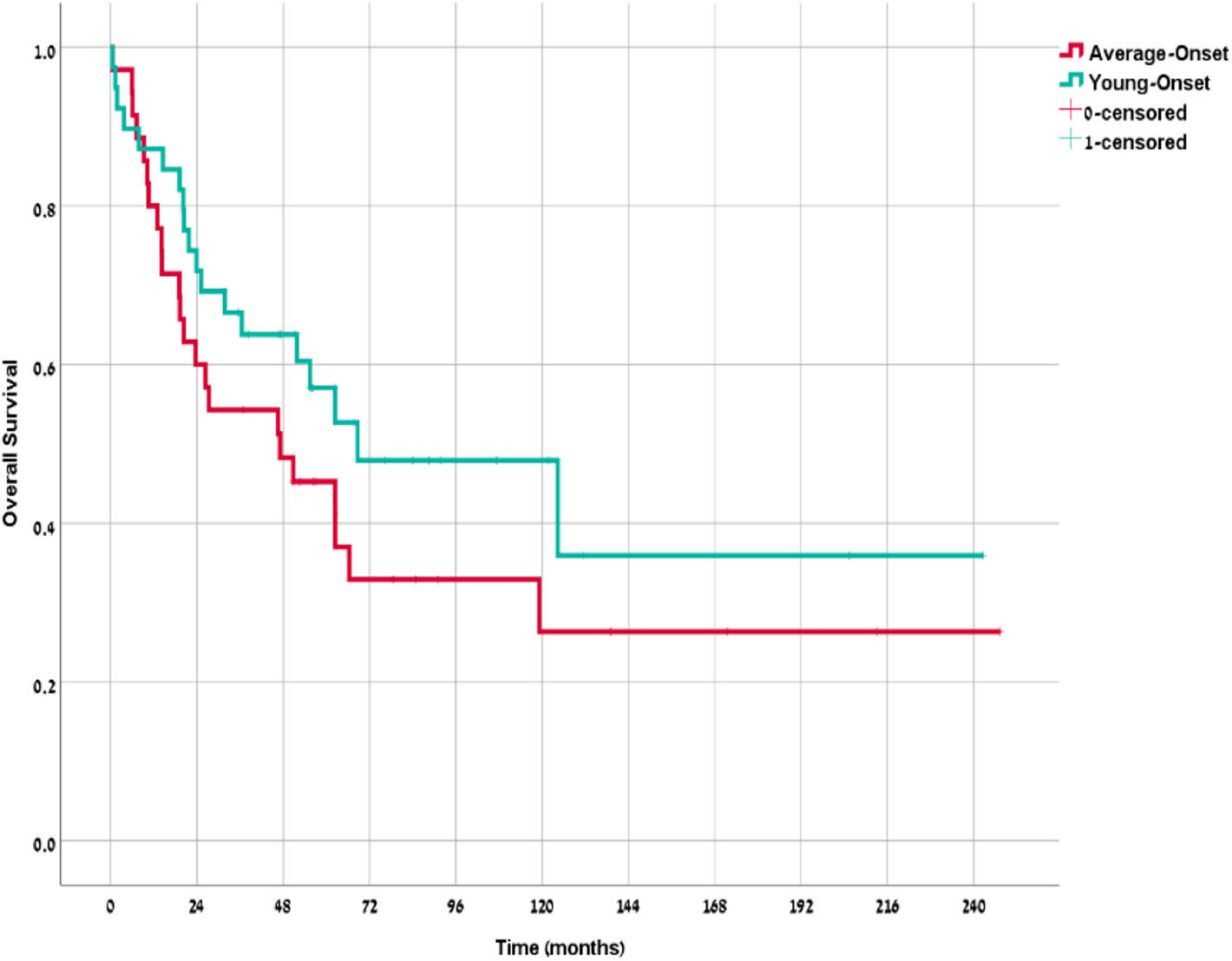
Overall Survival by Age Group

**Fig. 3:**
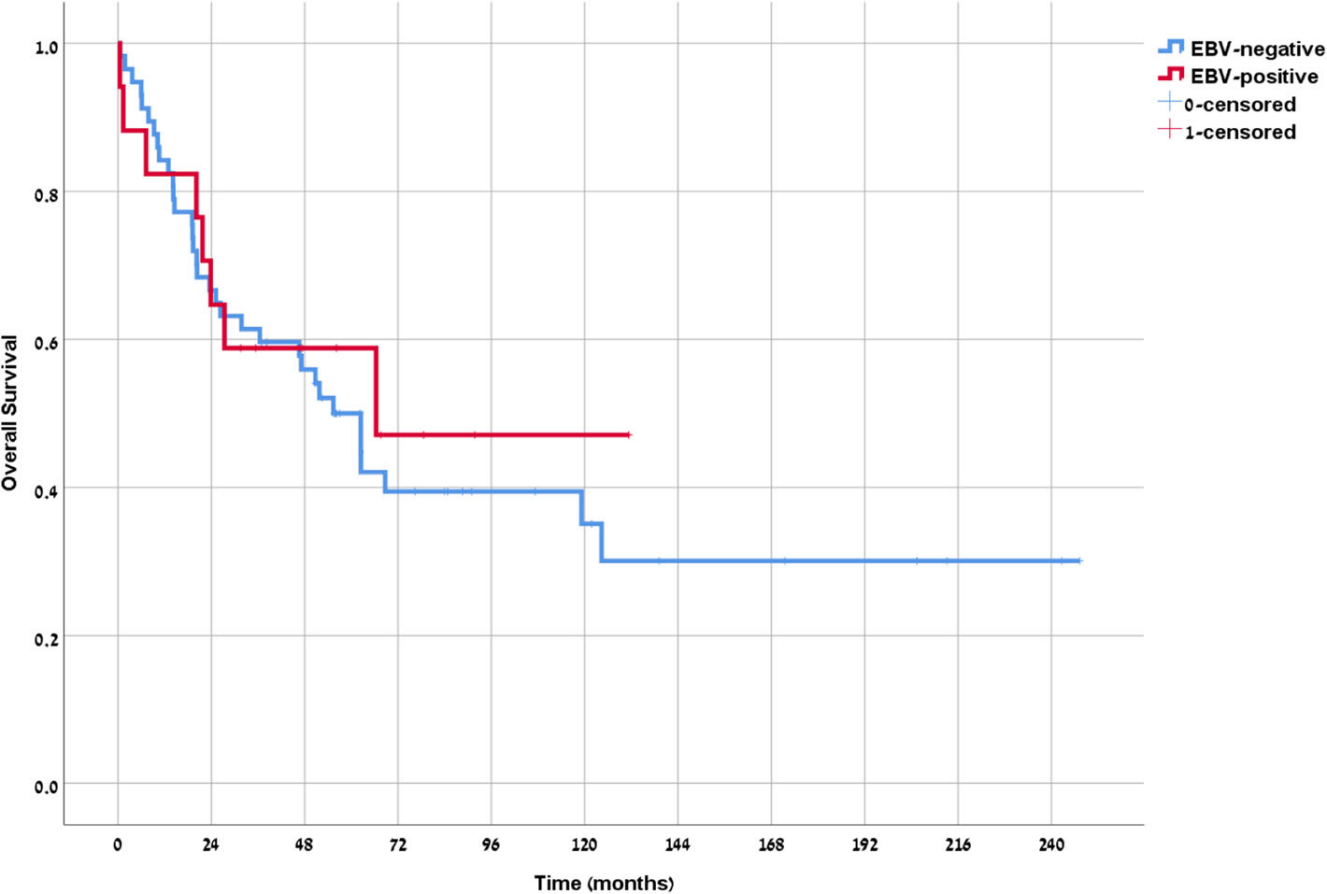
Overall Survival by EBV status Independent of Age

Median OS of the young-onset cohort was 69.7 months compared with 47.8 months in the average-onset cohort (*p* = 0.19) (Fig. 2). Median OS for EBV positive patients was 67.4 months compared with 56.2 months for EBV negative (*p* = 0.7) (Fig. 3). Median OS for the young-onset patients who were EBV positive (*n* = 13) was not reached compared with 56.2 months for the remaining cohort (*p* = 0.47).

## Discussion

Recent advances in state of the art molecular technologies have yielded the TCGA characterization of the genomic landscape of GC into four subtypes: the EBV-positive subtype, microsatellite unstable tumors, genomically stable tumors, which are enriched for the diffuse histological variant and tumors with chromosomal instability [11]. We hypothesized that the pathogenesis of young-onset GC may be attributed in part to environmental factors, and that the EBV subtype may be more prevalent in the young-onset population. Our results support our hypothesis, whereas in the young-onset cohort the EBV subtype was three-fold more prevalent (33% vs 11%, *p* = 0.025) than in the average-onset cohort, manifesting enhanced EBV and PD-L1 positivity. In our report, the prevalence of family history of malignancy did not vary between the age groups, although small sample size may affect this finding. This may support the concept that genetic factors are not dominant in young-onset GC.

Former evidence demonstrated that EBV associated GC occurs more frequently in male patients, most are intestinal type, tends to arise in the cardia or the body of the stomach [12]. A study that investigated the relationship between the TCGA subtypes and outcomes, found that the EBV subtype was associated with the best prognosis [13]. These patients seem to have a lower incidence of lymph node metastases, hence a lower tumor-node-metastasis system-stage and a better prognosis in terms of DFS and cancer-related survival [12]. A meta-analysis has reported conflicting results regarding the association between EBV positivity and age at diagnosis [7]. However, age was not reported in these studies. To the best of our knowledge, this is the first study to compare the differential age-dependent prevalence of the EBV subtype. This finding could have clinical implications.

There is former evidence implying that the presence of the EBV viral genome in gastric carcinomas may serve as a surrogate marker for targeted therapy. Gemcitabine was found to be a lytic inducer via activation of the ataxia telangiectasia-mutated (ATM)/p53 genotoxic stress pathway in EBV associated GC [14]. The efficiency of a gemcitabine- ganciclovir combination in EBV associated GC was established in a mouse model [14]. A study showed that EBV-positive GC have significantly greater expression of MHC class II on the tumor cells and a more extensive infiltrate of activated CD8+ T cells. This finding was most abundant in EBV-positive tumors that did not metastasize to lymph nodes. In addition, in EBV-positive GC without metastases, the infiltrate contained higher numbers of mature dendritic cells [15]. The TCGA has described a recurrent amplification at 9p24.1 at the locus containing *CD274* and *PDCD1LG2,* encoding PD-L1 and PD-L2. 9p amplifications were enriched in the EBV subgroup (15% of tumors). Evaluation of mRNA revealed elevated expression of *JAK2*, *PD-L1* and *PD-L2* in amplified cases [11]. A recent study has demonstrated PD-L1 staining in tumor cells in 50% (16/32) and immune cells in 94% (30/32) of Epstein-Barr Virus (EBV) + GCs cases. Among EBV-negative GCs, PD-L1 expression within tumors cells was observed only in cases with microsatellite instability (MSI), although 35% of EBV−/MSS GCs possessed PD-L1 expression of inflammatory cells [9]. Our study indicates that young-onset GC is characterized by both EBV positivity as well as enhanced immunostaining of PD-L1. Thus, our results may imply that this unique patient population may be a candidate for immunotherapy, which is an emerging novel treatment option in GC. Yet, to date, the surrogate biomarkers for patient selection for immunotherapy remain to be further elucidated. Nivolumab, a PD1 inhibitor, has shown efficacy and a survival advantage in unselect advanced GC after two or more previous chemotherapy regimens [16]. Pembrolizumab, a PD1 inhibitor, achieved a response rate of 26% in PD-L1 positive tumors as single agent in the first line setting [17]. An interesting question is whether the viral carrier status is an independent marker for response to immunotherapy regardless of PD-L1 status. In a phase I/II study, nivolumab is evaluated as monotherapy and in combination with chemotherapy against virus associated cancers, including EBV associated GC [18]. In an early update that included 24 patients with gynecological malignancies, the overall response rate was 21% [19]. An interesting finding was that in the average-onset cohort, all MSI-H patients were PD-L1 negative. This finding is unlike other reports indicating a correlation between MSI and PD-L1 positivity. Whether this is related to sample size or differences between populations, is unknown.

There have been several attempts to define the correlation between EBV and HER2. In this report, all EBV positive cases were HER2 negative, compared with 9% HER2 positivity in EBV negative cases (*p* = 0.23). One study has found significantly less high HER2 expression in EBV positive cases than in EBV negative (5% versus 24%; *p* < 0.001) and has suggested that LMP2A may suppress the HER2 expression [20]. Other studies have also found a negative correlation between EBV and HER2 positivity [21, 22]. One study has found a slightly higher HER2 expression in EBV positive GC cases (7%) than described in other reports [23]. When considering the 22% HER2 overexpression in the ToGA trial [24], it seems that HER2 is negatively correlated with the EBV subtype.

An interesting observation is that while significantly more young-onset patients were diagnosed with metastatic disease (27% vs 9%, *p* = 0.0498), overall survival was 69.7 vs 47.8 months in the young-onset and average-onset cohort, respectively (*p* = 0.19). The seemingly equivalent or better results despite the higher proportion of advanced stage patients for the young onset is likely derived from the more prevalent EBV subtype, as there was no difference in the proportion of patients undergoing curative resection, in DFS after surgery and median number of systemic treatment lines for metastatic disease between the cohorts.

This study is limited by its retrospective design, which is generally associated with methodological biases and difficulties in results interpretation. The most concerning bias in our study is clearly associated with patient selection. Yet, we tried to minimize selection bias by including all consecutive young patients in a period of 20 years, for whom we had tissue samples. The other limitation is not to have full sequence of the tumors to better characterize all four subtypes. The number of patients in each cohort is relatively low, and thus limits statistical robustness. The young-onset cohort contains two patient populations, German and Israeli. These populations may have unique attributes that would have been found uncovered if the cohorts were larger, and influence results interpretation. Also, having been treated in two cancer centers in different countries, means there may have been inhomogeneity with regard to patient work-up and treatment.

Nevertheless, despite the study limitations and small sample size, some results were significant and have paved us the way to design an ongoing prospective study to characterize the microbial factors of GC in young-onset and average-onset patients, such as EBV, *H. pylori*, Hepatitis B, along with histological biomarkers and immune factors (imunoscore®).

We aim to assess the relationship between EBV antibody titer, specific EBV strains, other causative factors and the risk of GC. This could lead to the identification of a high-risk group of patients that may benefit from screening for GC.

## Conclusion

The association between EBV positivity and GC in this study suggests that EBV may play a key role in the pathogenesis of young-onset GC. Since young-onset GC is not predominated by hereditary factors, environmental and microbial factors should be further studied as essential contributors, what may potentially govern early detection in high risk populations. Determining the prevalence of EBV associated GC in young patients could elucidate the feasibility of future targeted therapy for this unique patient group. Establishing a distinct clinical approach may eventually improve outcomes. It may very well be that in the future, young patients will be screened for PD-L1 and EBV positivity for optimal treatment selection. We are currently evaluating our findings in a prospective trial.

## Data Availability

The datasets used and/or analyzed during the current study are available from the corresponding author on reasonable request.
